# Functional Characterization of Colon-Cancer-Associated Variants in *ADAM17* Affecting the Catalytic Domain

**DOI:** 10.3390/biomedicines8110463

**Published:** 2020-10-30

**Authors:** Jan Philipp Dobert, Anne-Sophie Cabron, Philipp Arnold, Egor Pavlenko, Stefan Rose-John, Friederike Zunke

**Affiliations:** 1 Institute of Biochemistry Christian-Albrechts-Universität zu Kiel, 24118 Kiel, Germany; stu118262@mail.uni-kiel.de (J.P.D.); acabron@biochem.uni-kiel.de (A.-S.C.); stu119549@mail.uni-kiel.de (E.P.); rosejohn@biochem.uni-kiel.de (S.R.-J.); 2 Institute of Anatomy, Christian-Albrechts-Universität zu Kiel, 24118 Kiel, Germany; p.arnold@anat.uni-kiel.de; 3 Department of Molecular Neurology, University Hospital Erlangen, Friedrich-Alexander-Universität Erlangen-Nürnberg, 91054 Erlangen, Germany

**Keywords:** ADAM17, colorectal cancer (CRC), TNFα, IL-6R, AREG, shedding

## Abstract

Although extensively investigated, cancer is still one of the most devastating and lethal diseases in the modern world. Among different types, colorectal cancer (CRC) is most prevalent and mortal, making it an important subject of research. The metalloprotease ADAM17 has been implicated in the development of CRC due to its involvement in signaling pathways related to inflammation and cell proliferation. ADAM17 is capable of releasing membrane-bound proteins from the cell surface in a process called *shedding.* A deficiency of ADAM17 activity has been previously shown to have protective effects against CRC in mice, while an upregulation of ADAM17 activity is suspected to facilitate tumor development. In this study, we characterize ADAM17 variants found in tissue samples of cancer patients in overexpression studies. We here focus on point mutations identified within the catalytic domain of ADAM17 and could show a functional dysregulation of the CRC-associated variants. Since the catalytic domain of ADAM17 is the only region structurally determined by crystallography, we study the effect of each point mutation not only to learn more about the role of ADAM17 in cancer, but also to investigate the structure–function relationships of the metalloprotease.

## 1. Introduction

Cancer is one of the leading causes of death in modern society. In 2019, over 1.7 million new cases and over 600,000 deaths were reported in the US alone. Colorectal cancer (CRC) cancer is among the more common and also lethal forms of it, being ranked third in terms of incidence for both males and females [1]. The highest non-genetic risk factor for developing CRC is a chronic inflammation of the gut, generally known as inflammatory bowel disease (IBD) [2,3], which includes a subset of diseases resulting in chronic inflammation of the gastrointestinal tract, such as ulcerative colitis or Crohn’s disease [4]. Although many susceptibility genes for IBDs are known, most cases can be linked to a variety of different environmental factors, including diet, smoking, stress and many more [5]. A so-called “Western diet” (high fat, low fiber) has been associated with a higher risk of developing IBD and CRC in humans and mice alike [6,7,8]. In recent years, the membrane-bound metalloprotease ADAM17 (a disintegrin and metalloprotease 17) has been identified as a key player in the development of colon cancer. First discovered as the protease responsible for cleaving proTNFα at the cell surface [9], ADAM17 has since been found to cleave over 80 substrates and thus being involved in almost all parts of cell homeostasis [10]. This includes inflammation, regeneration, differentiation and immunity. Underlining the important role of ADAM17 in vivo, *ADAM17* knock out mice are not viable and die prenatally [11]. A hypomorphic mouse model expressing very low levels of ADAM17 (~5%) is viable, but highly compromised [12]. Due to its wide range of functions and its almost ubiquitous expression throughout the organism, a pathophysiological role of ADAM17 has long been implicated [13]. ADAM17 is expressed as a zymogen and consists of six major domains: an inhibitory pro-domain that also functions as a chaperone [14], a catalytic domain (CD), a disintegrin domain, a membrane-proximal domain harboring the CANDIS region [15], a transmembrane domain and a small cytosolic tail. During the maturation process, the pro-domain is cleaved by furin in the trans-Golgi network, exposing the catalytic site and enabling protease activity [16]. ADAM17 is then transported to the cell surface where it acts as sheddase [10].

In addition to activation by furin, regulation of ADAM17 can take place by phosphorylation of the cytoplasmic domain [17,18,19,20], cellular localization [21], composition of the cell membrane [22] and/or activation/inactivation by protein-disulfide isomerase [23]. Another regulatory mechanism is substrate recognition, which is thought to be facilitated mainly by the membrane-proximal domain. In addition, the transmembrane proteins iRhom1 and iRhom2 have been found to be crucial for ADAM17 substrate selectivity, trafficking and activation [24,25,26,27]. Other studies have shown that even after maturation, the cleaved pro-domain can function as a potent inhibitor of ADAM17 [28]. Given its importance in such fundamental cellular pathways, it is no surprise that ADAM17 is highly regulated by so many different mechanisms. Interestingly, no protein structure of full-length ADAM17 has been obtained yet. Single domain structures have been solved only for the catalytic domain [29] as well as the membrane-proximal domain [23]. Among the many signaling pathways ADAM17 is involved in, the most prominent ones are connected to inflammation and regeneration. In inflammation, ADAM17 is responsible for the release of TNFα and its receptors TNFR1 and TNFR2, which are essential for an inflammatory response [30]. High levels of soluble TNFα are associated with an inflammatory state and are a hallmark of IBD [31]. Many other proteins involved in leukocyte activation are also shed by ADAM17, such as L-selectin, VCAM-1, ICAM-1 and more [32,33,34,35]. ADAM17 also influences the Interleukin 6 (IL-6) pathway by shedding the Interleukin 6 receptor (IL-6R) from the cell surface [36]. IL-6-mediated pathways are also heavily involved in inflammation, acting in both a pro- and anti-inflammatory manner [37,38]. Almost all inflammatory diseases are associated with an upregulation of IL-6. Signaling can be induced in two different way, typically described as classic and trans-signaling. In classic signaling, IL-6 binds to IL-6R at the cell surface, then a gp130 homodimer is recruited to form the signaling complex [39]. However, if IL-6R has been shed from the cell surface, IL-6 can still bind to the shed ectodomain of IL-6R, forming a soluble ligand–receptor complex. This complex can then bind to a gp130 homodimer on any type of cell (even those not expressing IL-6R) to form the trans-signaling complex [40]. While classic IL-6 signaling is associated with anti-inflammatory properties, trans-signaling has been shown to have a pro-inflammatory effect and play a major role in the development of cancer [41,42,43].

In regeneration, ADAM17 is capable of regulating cell proliferation by cleaving and releasing EGF-R ligands like Amphiregulin (AREG), TGF-α, Hb-EGF and EREG [44]. The EGF receptor (ErbB1) is a transmembrane tyrosine kinase receptor capable of binding different ligands [45]. Activation of EGF-R can induce a variety of signaling cascades involved in cell survival, proliferation and differentiation [46]. EGF-R overexpression, as well as increased activation of EGF-R pathways, are high risk factors for developing cancer and EGF-R signaling is often highly upregulated in human carcinomas [47,48,49]. Previous studies have shown that ADAM17 is upregulated in colon tumor tissue [50]. In mice, ADAM17-deficiency has a protective effect on tumor burden in an induced genetic colon cancer model [51]. Thus, ADAM17 activation seems to play a key role in the development of CRC. The specific role of ADAM17 in CRC, however, appears to be multifaceted as all three major ADAM17-regulated pathways (TNFα-, EGF-R- and IL-6-signaling) on different cell types are implicated in tumor progression. A recent study has found that both the activation of EGF-R pathways, as well as IL-6 trans signaling can be observed during tumor development, and both require ADAM17 [51]. Release of sIL-6R by ADAM17 to promote IL-6 trans-signaling can be facilitated by tumor cells and macrophages [52,53], whereas ADAM17 on myeloid cells is speculated to release EGF-R ligands and activate EGF-R signaling in an autocrine manner, as well as on macrophages. This in turn leads to the production of IL-6, further promoting IL-6 trans-signaling [43,54]. 

In this study, we analyzed naturally occurring mutations within the *ADAM17* gene found in colon cancer tissues, utilizing databases (IntOGen, COSMIC, TCGA and ICGC) [55,56,57,58,59,60] containing sequence data of patient tumor samples. Interestingly, single nucleotide variations (SNVs) could be found distributed all over the *ADAM17* gene and are hence found within all protein domains of the metalloprotease. 

We here selected three ADAM17 missense point mutations from colon cancer tissue (E319G, E406X, M435I) as well as one variant found within pancreatic cancer (P417Q). All these variants are found within the catalytic domain of the translated protein. The E406X mutation is located right at the beginning of the Zn^2+^ binding motif and introduces an early stop codon. The resulting truncated protein thus consists of only the pro-domain and a small part of the catalytic domain. Out of the four selected mutants, only the M435I variant has been subject to research before. This variant was found to be catalytically inactive as the methionine at this position is part of a highly conserved loop structure integral for enzymatic function [61]. Utilizing an ADAM10/17 deficient HEK293 cell line, we characterized these ADAM17 variants based on their expression, cellular localization as well as their ability to cleave substrates implicated in cancer.

## 2. Experimental Section

### 2.1. Database Analysis

The ADAM17 variants were found by screening for cancer-associated mutations in the catalytic domain. The databases used are the following: IntOGen (Integrative Onco Genomics, Barcelona Biomedical Genomics Lab, Barcelona, Spain), Cosmic (Catalogue of Somatic Mutations in Cancer; Sanger Institute, Cambridge, UK), The Cancer Genome Atlas Program (TCGA; National Institutes of Health, Bethesda, MD, USA) and the International Cancer Genome Consortium (ICGC). These databases provide somatically acquired mutations found in tumor tissue of cancer patients (the project identification code, Date Month Year). 

### 2.2. cDNA Constructs and Cloning

Expression plasmids of murine (m) ADAM17 wild type (wt), as well as the colon cancer-associated mutants (E319G, E406X, M435I, P417Q), were assembled using mADAM17 cDNA and the pcDNA3.1 (+) vector (#V79020, Thermo Fisher Scientific Inc., Waltham, MA, USA). Mutations were introduced via site-directed mutagenesis PCR. The generated constructs were verified by using Sanger sequencing (Eurofins Genomics, Ebersberg, Germany). Expression plasmids of ADAM17 substrates (murine AREG, IL-6R and proTNFα) were also based on pcDNA3.1 (+) and were used for co-transfection experiments in order to analyze ADAM17 enzymatic activity. For staining of the plasma membrane in immunofluorescence analysis (see Section 2.7), a custom made eGFP construct was used. It comprises the coding sequence of eGFP with an added farnesylation motif for membrane anchoring, cloned into pcDNA3.1 (+).

### 2.3. Cell Culture

HEK cells deficient for ADAM10 and ADAM17 (A10/A17 dKO) were used for overexpression experiments as recently described [14]. Cells were cultivated in Dulbecco’s Modified Eagle’s medium (DMEM; #D6429, Sigma-Aldrich, Munich, Germany) supplemented with 10% heat-inactivated fetal calf serum (FCS) (#3306-PI31004, PAA Laboratories, Pasching, Austria) and 1× penicillin/streptomycin (Pen/Strep; #P0781, Sigma-Aldrich, Munich, Germany). Passaging of the cells was required every 3–4 days once they reached a confluency of 80–90%.

### 2.4. Transfection

Prior to transfection, the cell count of the respective cell line was determined via Cellometer Auto T4 Plus (Nexcelom Bioscience, Lawrence, MA, USA). The HEK A10/17 dKO cells were then plated at a density of 2.2 × 10^6^ per 10 cm plate and 2 × 10^5^ per 6-well and cultivated overnight. The next day, cells were transfected with a mixture of polyethylenimine (PEI, 1 µg/µL) (#24765-1, Polysciences, Warrington, PA, USA) and DNA (1 µg/µL) in a ratio of 3:1 diluted in DMEM. Per plasmid, 1 µg of DNA was used for transfection of a 10 cm dish and 0.5 µg of DNA per well of a 6-well plate. The cells were harvested after 48 h and, where indicated, stimulated with 200 nM phorbol 12-myristate 13-acetate (PMA; #P8139, Sigma-Aldrich, Munich, Germany) for 2 h prior to harvesting.

### 2.5. Western Blot Analysis

HEK A10/A17 dKO cells were transfected as described above and harvested by mechanically detaching from the culture dish. Afterwards, the cells were washed twice with cold PBS and lysed in lysis buffer (1% Triton X-100, 150 mM NaCl, 50 mM Tris-HCl pH 7.4), 10 mM 1.10-phenanthroline (#841491, Merck, Darmstadt, Germany) and 1× cOmplete protease inhibitor cocktail (#11697498001, Roche, Basel, Switzerland) for 60 min at 4 °C. The protein concentration was determined by BCA assay (#23225, Thermo Fisher Scientific Inc., Waltham, MA, USA). Then, 40 µg of total protein was supplemented with 5× SDS sample buffer (0.3 M Tris-HCl, pH 6.8, 10% SDS, 50% glycerol, 5% β-mercaptoethanol, 5% bromophenol blue) and denatured at 95 °C for 5 min. The samples were then run in an SDS-PAGE and transferred to a PVDF-membrane (#IPFL00010, Merck Millipore, Burlington, MA, USA). The membrane was blocked in TBS containing 6% milk powder and incubated with primary antibodies overnight at 4 °C. Secondary antibodies were applied for 1 h at room temperature. 

The following primary antibodies were used: anti-ADAM17 10.1 (binding epitope between aa 290−309; Pineda Antikörper-Service, Berlin, Germany), anti-TNFα (Pineda Antikörper-Service, Berlin, Germany), anti-IL-6R C#1 (Pineda Antikörper-Service, Berlin, Germany), anti-myc (Cell Signaling, Frankfurt am Main, Germany; clone 9B11 #2276), anti-transferrin receptor (#ab84036, abcam, Cambridge, UK) and anti-β-actin (#A5441, Sigma-Aldrich, Darmstadt, Germany). As secondary antibodies, goat anti-rabbit HRP and sheep anti-mouse HRP (#111-035-144, #515-035-062, both Dianova, Hamburg, Germany), as well as IRDye 800CW donkey anti-rabbit and IRDye 680RD Donkey anti-Mouse (#926-32213, #926-68072, both LI-COR Biosciences, Lincoln, Nebraska) were used.

### 2.6. Biotinylation

First, 2 × 10^6^ HEK A10/A17 dKO cells were transfected as described above. Twenty-four hours after transfection, the cells were cooled to 4 °C, washed twice with ice-cold PBS-CM (PBS with 0.1 mM CaCl_2_ and 1 mM MgCl_2_ added) and afterwards incubated with 1 mg/mL Sulfo-NHS-SS-Biotin (#21331, Thermo Fisher Scientific Inc., Waltham, MA, USA) in PBS-CM for 30 min at 4 °C. Simultaneously, control cells were incubated in only PBS-CM. The solutions were removed and ice-cold quenching buffer (PBS plus 50 mM Tris-HCl; pH 8.0 in PBS-CM) was added for 10 min. After three washing steps with PBS-CM the cells were lysed in biotinylation lysis buffer (1% Triton X-100, 0.1% SDS, 150 mM NaCl, 50 mM Tris-HCl pH 7.4 and 1× complete protease cocktail inhibitor) for 30 min at 4 °C. The protein amount was determined by BCA and an aliquot of 20 µg of total protein was taken as a lysate control sample and prepared for SDS-PAGE. An equal amount of total protein from each sample was then diluted to a volume of 250 µL using biotinylation lysis buffer. Then, 75 µL of streptavidin beads (#20359, Thermo Fisher Scientific Inc., Waltham, MA, USA) per sample were washed three times with 100 µL biotinylation lysis buffer and added to the diluted samples. After an incubation of 1 h at 4 °C, the supernatant was removed and the beads were washed several times with 500 µL of biotinylation lysis buffer. Afterwards, all supernatant was removed and 40 µL 1× SDS sample buffer were added to the beads of each sample and heated up to 60 °C for 20 min. All samples were then analyzed via SDS-PAGE and Western blot.

### 2.7. Immunofluorescence Analysis

HEK A10/A17 dKO cells were seeded in a 6-well plate (2.0 × 10^5^ cells per well) containing glass cover slips and transfected as previously described. The cells were then washed with PBS, fixed with 4% PFA in PBS for 15 min at room temperature and permeabilized with 0.3% Triton X-100 (Sigma-Aldrich, Darmstadt, Germany) in PBS for 30 min. Subsequently, cells were blocked in blocking buffer (2% BSA, 5% heat-inactivated FCS and 0.3% Triton in PBS) for 60 min and then incubated at 4 °C overnight with the primary antibodies. All antibodies were diluted in blocking buffer to their working concentrations (see below). Afterwards, the cells were washed three times with PBS containing 0.3% Triton X-100, then incubated with secondary antibodies for 60 min at room temperature. Finally, cells were washed three more times with 0.3% Triton X-100 in PBS, once with PBS and stained with DAPI as part of the mounting mix consisting of Dabco (Sigma-Aldrich, Darmstadt, Germany) and Mowiol (Merck Millipore, Burlington, MA, USA). Immunofluorescence analyses were performed with a confocal laser scanning microscope (FV1000, Olympus, Tokyo, Japan) equipped with a U Plan S Apo 100× oil immersion objective. Digital images were analyzed using FV10-ASW Viewer version 4.2 (Olympus, Tokyo, Japan). Primary antibodies used: anti-PDI (1:100, abcam, Cambridge, UK; #ab13506), anti-ADAM17 10.1 (1:100, Pineda Antikörper-Service, Berlin, Germany). Secondary antibodies were purchased from Thermo Fisher Scientific (Waltham, MA, USA): goat-anti-mouse Alexa Fluor 594 (#A11032) and goat-anti-rabbit Alexa Fluor 488 (#A11037).

### 2.8. Enzyme-Linked Immunosorbent Assay (ELISA)

First, 3.5 × 10^5^ cells were transfected as previously described with each ADAM17 variant and the respective substrate (mAREG, mIL-6R or mpro-TNFα). The shed cytokines were determined in the cell-free supernatant by ELISA according to the manufacturer’s instructions (mTNFα: #11560637 eBioscience, Frankfurt am Main, Germany; mIL-6R and mAREG: #DY1830 and #DY989, R and D Systems, Minneapolis, MN, USA).

### 2.9. Live Cell Surface ADAM17 Activity Assay

First, 2 × 10^6^ HEK A10/A17 dKO cells were seeded onto a 10 cm culture dish and transfected the next day. Twenty-four hours after transfection the cells were detached from the culture dish using trypsin and seeded onto a 96-well plate (2 × 10^5^ cells per well). The following day, ADAM17 surface activity was measured by removing the culturing media and supplying the cells with PBS containing 20 µM of a quenched fluorogenic peptide (Abz-LAQAVRSSSR-Dpa; ADAM17 (TACE) substrate IV (Calbiochem, Merck, Darmstadt, Germany; #616407)). The fluorescence (λ_Ex_: 320 nm; λ_Em_: 405 nm) was measured every 30 s over a total of 120 min using a Tecan plate reader (Tecan Infinite 200 Pro, TECAN, Männedorf, Switzerland). ADAM17 surface activity was represented by the area under curve (fluorescence over time) and normalized to wt.

### 2.10. Structural Analysis

The catalytic domain of ADAM17 was visualized based on the crystal structure published by Mazzola et al. (PDB: 3E8R) [29]. Molecular graphics and analyses were performed with UCSF Chimera (version 1.14), developed by the Resource for Biocomputing, Visualization, and Informatics at the University of California, San Francisco, with support from NIH P41-GM103311 [62]. Solvent-excluded molecular surfaces were created with the help of the MSMS package [63].

### 2.11. Data Analysis and Statistic

All values are expressed as the mean ± SEM. For data analysis Excel (Microsoft, Redmond, WA, USA) and GraphPad Prism version 7 (GraphPad Software, San Diego, CA, USA) were used. Differences among mean values were analyzed by two-tailed, unpaired Student t test or one-way ANOVA, followed by Tukey’s multiple comparison test where applicable. In all analyses, the null hypothesis was rejected at *p* < 0.05 with * < 0.05, ** < 0.01, *** < 0.001, **** < 0.0001.

## 3. Results

### 3.1. Cloning and Expression of ADAM17 Mutations

We searched the databases IntOGen, COSMIQ, TCGA and ICGC for mutations within the *ADAM17* gene. These databases are listing somatic mutations found in tumor tissue of cancer patients. The search for ADAM17 came up with 175 results of single nucleotide variations (SNVs) within unique cancer tissue samples (Figure S1A). In this study, we focused on missense point mutations identified in colon cancer samples (in total 11 different ADAM17 variants were found; Figure S1B). Interestingly, colon cancer-associated point mutations are distributed over the whole protein, located in following domains: pro-, catalytic-, disintegrin-, membrane proximal and cytoplasmic domain (Figure S1B). Most variants were found within the catalytic and membrane proximal domain (both three different mutations; Figure S1B), underlining the importance of both domains for proper enzymatic function.

**Figure 1:**
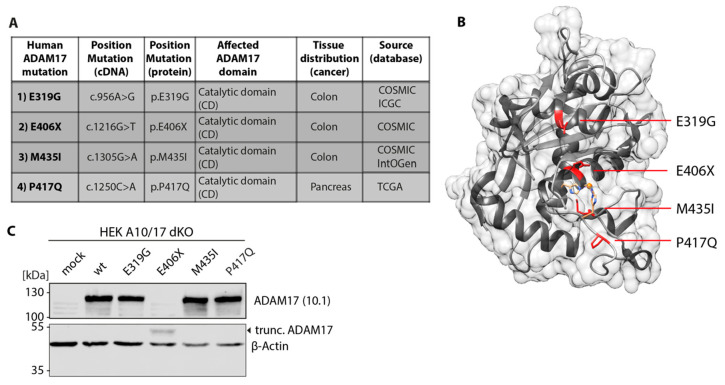
Overview and expression of cancer-associated ADAM17 variants within the catalytic domain. (**A**) Table and description of somatic mutations in human ADAM17 found within tumor tissue of colon and pancreatic cancer patients. Listed is also the position in the cDNA and amino acid sequence as well as the database (COSMIQ, ICGC, IntOGen or TCGA) in which there were found. (**B**) Structural model of catalytic domain (CD) of ADAM17 including mutations highlighted in red (structure derived from PDB: 3E8R). The three histidine residues coordinating the zinc ion in the active center are also highlighted in orange. (**C**) Overexpression of ADAM17 variants in ADAM10/ADAM17 double-deficient HEK cells (HEK A10/A17 dKO). Representative immunoblot showing equal protein levels of ADAM17 wild type (wt) and the ADAM17 variants after overexpression. Only the truncated variant E406X (black arrow, ~50 kDa) exhibits a lower expression level. β-actin was used as a loading control.

We here analyzed four cancer-associated missense mutations located within the catalytic domain of the ADAM17 protein (Figure 1A). First, we focused on the point mutation E319G (c.956A>G; p.E319G) found in tumor tissue of colon cancer patients. The negatively charged glutamic acid at position 319 is replaced by a glycine, which is located in an α-helix offside the active center (Figure 1B). Next, the colon cancer-associated E406X (c.1216G>T; E406X) mutation was studied (Figure 1A). The mutation leads to a premature stop after His405, resulting in a truncated ADAM17 protein lacking the last 422 amino acids, which includes the three histidines that form the active center (Figure 1B). The M435I (c.1305G>A; p.M435I) variant, which was also found in colon cancer samples, was the third mutation analyzed in this study (Figure 1A). Here, the methionine of the Met-turn structure near the active site is replaced by an isoleucine (Figure 1B). Last, the P417Q (c.1250C>T; p.P417Q) variant was examined in this study. Its point mutation is located right next to the histidines (His405, His409 and His415) of the active center and the coordinated zinc ion (Figure 1B). In comparison to the other mutants, this variant was found in pancreatic tumor tissue and was included to this study because of its unique location within the catalytic domain.

All ADAM17 variants were generated by mutagenesis PCR and inserted into a pcDNA3.1 expression plasmid containing the mADAM17 cDNA. Afterwards, ADAM10 and ADAM17 double deficient HEK cells (HEK A10/17 dKO) were reconstituted with the ADAM17 variants and the wild type by transient overexpression. The expression was analyzed via SDS-PAGE and Western blot using an anti-ADAM17 antibody (10.1 antibody) (Figure 1C). The epitope region recognized by this antibody is still intact in the truncated ADAM17 variant E406X, hence this variant can be detected as a smaller protein at ~50 kDa. However, the E406X variant showed decreased protein levels compared to the other analyzed variants and the wild type (Figure 1C). This suggest stable protein expression of the variants E319G, M435I and P417Q, but not E406X. This truncation probably results in an unstable ADAM17 protein, making it prone for degradation processes.

### 3.2. Proteolytic Activity of Cancer-Associated ADAM17 Variants

**Figure 2:**
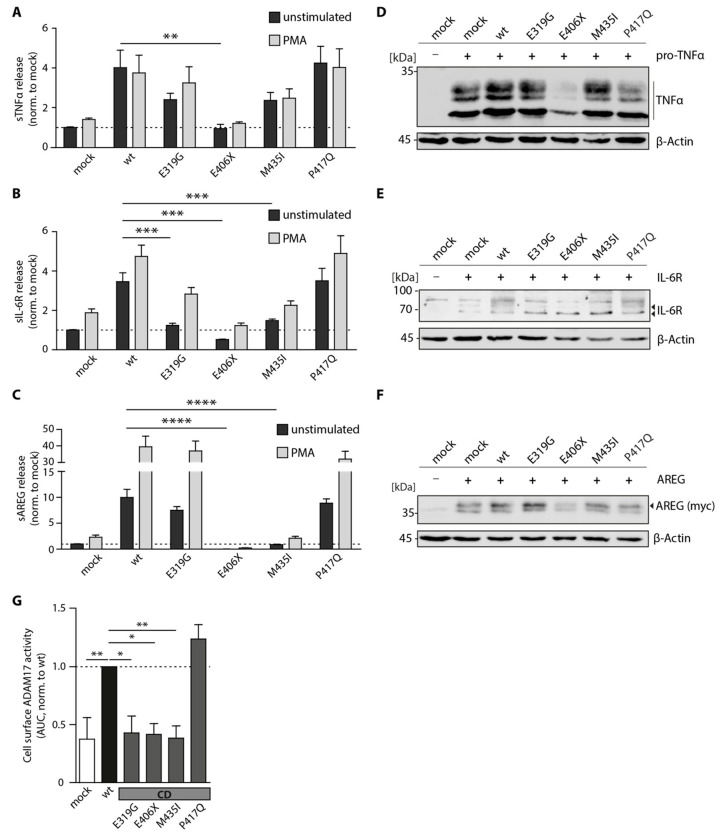
Proteolytic activity of ADAM17 variants. Enzyme-linked Immunosorbent Assay (ELISA) of soluble (s) TNFα (**A**), IL-6R (**B**) and AREG (**C**) measured in cell-free supernatants of HEK A10/17 dKO cells overexpressing ADAM17 wt and ADAM17 variants. Values have been normalized to mock transfected cells. Shown is the summary of three independent experiments statistically analyzed utilizing a one-way ANOVA together with a Tukey’s multiple comparison test. *** *p* < 0.005, **** *p* < 0.001. Representative immunoblots show expression of analyzed substrates TNFα (**D**), IL-6R (**E**) und AREG (**F**) in HEK A10/A17 dKO after co-transfection with ADAM17 variants. The here utilized AREG construct exhibits an N-terminal myc-tag, which was used for detection (anti-myc). β-actin was used as a loading control. (**G**) Cell surface activity assay of ADAM17 variants (wt and four mutations within the catalytic domain (CD): E319G, E406X, M435I, P417Q) in living HEK A10/A17 dKO. A quenched fluorogenic TNFα peptide was used and the increase in fluorescence was measured every 30 s for 120 min. Activity was determined as area under curve. Shown are normalized values (to mock) of three independent experiments. The statistical analysis was performed utilizing a one-way-ANOVA together with a Tukey’s multiple comparison test. * *p* < 0.05, ** *p* < 0.01, *** *p* < 0.001, **** *p* < 0.0001.

Next, we performed functional analyses of the aforementioned ADAM17 variants to study how the inserted point mutations affect the proteolytic activity of the enzyme. To do this, we co-transfected HEK A10/A17 dKO cells with respective ADAM17 variants along with one of the following ADAM17 substrates: pro-TNFα, IL-6 receptor (IL-6R) or amphiregulin (AREG). All of these substrates are shed by ADAM17 and the soluble (s) ectodomain is released into the cell supernatant. We then measured the amount of sTNFα (Figure 2A), sIL-6R (Figure 2B) and sAREG (Figure 2C) in the media utilizing ELISA assays. In addition, we stimulated the cells with the protein kinase C activator PMA (200 nM, 2 h), which is described to increase ADAM17-mediated shedding [36]. For HEK A10/A17 dKO cells transfected with wild type ADAM17, higher levels of sTNFα (Figure 2A), sIL-6R (Figure 2B) and sAREG (Figure 2C) could be measured in comparison to mock transfected cells. Interestingly, PMA treatment only resulted in increased shedding of IL-6R (Figure 2B), and AREG (Figure 2C), but not TNFα (Figure 2A). Equal protein expression of the substrates was verified via SDS-PAGE and Western blot: TNFα (Figure 2D); IL-6R (Figure 2E) and myc-tagged AREG (Figure 2F). All three co-transfected substrates together with the E406X variant showed slightly reduced protein expression (Figure 2D–F). The E406X variant also showed no activity towards any of the analyzed substrates measured by ELISA assay (Figure 2A–C). This was expected since this variant lacks the active center and zinc-binding motif. Interestingly, the variant E319G seemed to be partially active and exhibited shedding activity towards TNFα (Figure 2A) and AREG (Figure 2C). This variant only exhibited partial enzymatic shedding activity towards IL-6R when stimulated with PMA (Figure 2B). The M435I variant presented with significantly decreased enzymatic activity towards IL-6R (Figure 2C) and AREG (Figure 2E) even after PMA stimulation. Only the P417Q variant showed shedding activity towards all here analyzed substrates (TNFα, IL-6R, AREG) at comparable levels as the ADAM17 wild type (Figure 2A–C).

To further study the enzymatic activity of the ADAM17 variants, a live-cell surface activity assay of ADAM17 was performed by utilizing a quenched fluorogenic TNFα peptide. Intriguingly, all three colon cancer-associated mutants (E319G, E406X, M435I) showed diminished enzymatic activity on the cell surface comparable to the mock control, whereas the pancreatic cancer variant P417Q exhibited peptide cleavage even above ADAM17 wild type level (Figure 2G).

Taken together, these results show that the point mutation E319G, E406X and M435I in close proximity to the active center have a negative influence on the shedding activity of ADAM17. In contrast, the variant P417Q seemed to be as active as the wild type.

### 3.3. Cellular Localization of ADAM17 Variants

**Figure 3:**
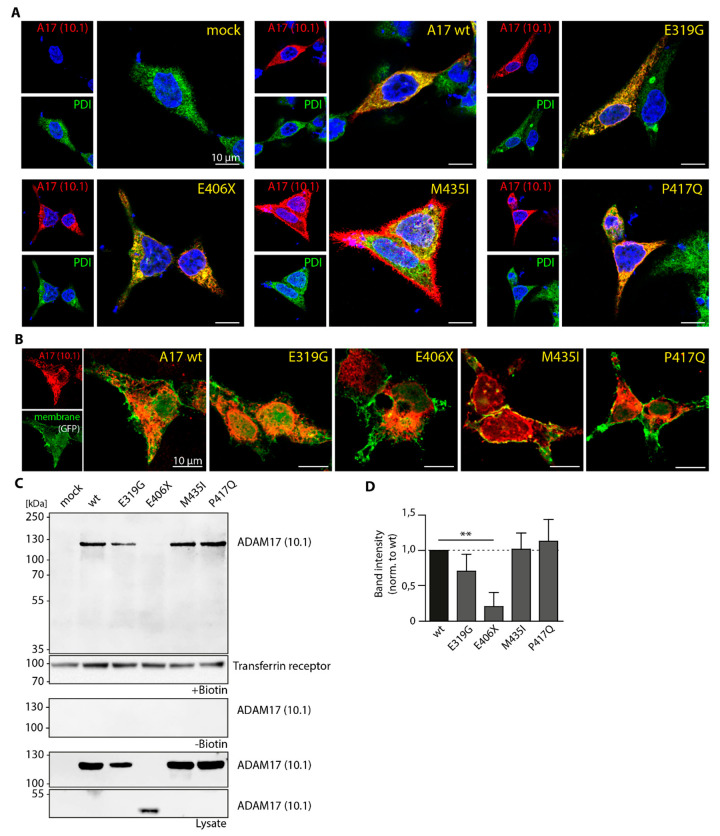
Cellular localization of ADAM17 variants. (**A**) Representative immunofluorescence pictures of HEK A10/A17 dKO cells transfected with the ADAM17 variants (red; antibody: 10.1). As reference, the ER was stained (green) using an anti-PDI antibody. Scale bar: 10 µm. (**B**) Representative immunofluorescence pictures of HEK A10/A17 dKO cells transfected with ADAM17 variants (red; antibody: 10.1), as well as a membrane targeted eGFP construct (green) to visualize ADAM17 on the cell surface. (**C**) Representative immunoblots of biotinylated HEK A10/A17 dKO cells transfected with the ADAM17 variants. The transferrin receptor was used as a control for positive pull-down of cell surface proteins. Also shown are HEK A10/A17 dKO incubated without biotin as a negative control. The lysate control (first and second to last blot) shows the input of the biotinylation assay. (**D**) Quantification of biotinylation. The values shown are derived from three independent experiment and normalized first to expression level in cell lysates and then shown relative to wt. A one-way-ANOVA together with Tukey’s multiple comparison test was performed. ** *p* < 0.01.

An impaired trafficking during maturation of the protein within the secretory pathway, for example between endoplasmic reticulum (ER) and cell surface could be an explanation for the decreased ADAM17 shedding activity of the variants E319G, E406X and M435I. Therefore, we performed immunofluorescence (IF) stainings of HEK A10/A17 dKO cells reconstituted with the respective variants (Figure 3A,B). In Figure 3A, ADAM17 (red) was stained using the anti-ADAM17 antibody 10.1. As reference the ER (green) was co-stained using an anti-PDI antibody. The wild type and the variant P417Q co-localized with the ER, but also partly appeared on the cell surface (Figure 3A) and seemed to reach the cell surface to a similar extent. The truncated variant E406X exhibited accumulation within the ER, indicated by a strong co-localization with PDI and the appearance of clumpy ER structures, suggesting impaired trafficking out of the ER. This seems to be also true for the E319G variant (Figure 3A). However, the accumulation of the E319G within ER structures was not as prominent as for the E406X variant. Interestingly, in some cells the M435I showed an excessive amount of ADAM17 outside of the ER (Figure 3A).

For better visualization of ADAM17 on the cell surface, a second immunofluorescence experiment was conducted (Figure 3B), in which HEK A10/A17 dKO cells were co-transfected with a membrane-targeted GFP construct (farnesylated eGFP, green channel) in addition to ADAM17 reconstitution (red). For the wild type, as well as the mutants E319G, M435I and P417Q, ADAM17 signal could be observed at the cell surface (Figure 3B). In comparison, the E406X variant appeared to localize towards the center of the cell with the ADAM17 signal not fully extending to the plasma membrane, further suggesting accumulation in the ER and no cell surface expression.

For quantitative analysis of ADAM17 cell surface transport, a biotinylation and pulldown of cell surface proteins from transfected HEK A10/A17 dKO cells was performed, followed by Western blot analysis (Figure 3C). As a positive control for the pulldown, we stained for the cell surface protein transferrin receptor. A biotin-negative control (- Biotin) was used to show specificity and sensitivity of the analysis (Figure 3C). Moreover, the lysate control confirmed similar input/expression of ADAM17 protein (Figure 3C). Except for the truncated E406X mutant, all here analyzed ADAM17 variants, including the wild type, were detectable at the cell surface. It seems that the E406X is not transported to the cell surface, as already indicated by the IF analysis (Figure 3A,B). This was expected as this variant lacks a transmembrane domain and therefore cannot be anchored to the membrane. To quantify the amount of ADAM17 on the cell surface, the band intensity of each of the biotinylated samples was normalized to the respective lysate control and is expressed relative to the wild type (Figure 3D). The E319G, P417Q and M435I variant showed a similar band intensity to the wild type after normalization, indicating that all three reach the cell surface.

Overall, our data indicate that colon cancer-associated ADAM17 variants (E319G, E406X, M435I) exhibit diminished substrate recognition and/or enzymatic activity towards physiological substrates of the metalloprotease (TNFα, IL-6R, AREG). Although the E319G and M435I variants were found to be localized at cell membrane, they did not show any activity on the cell surface towards a fluorogenic TNFα peptide. This indicates that both point mutations within the catalytic domain do not influence intracellular protein trafficking pathways, but rather affect enzymatic function. Intriguingly, the pancreatic cancer-associated variant P417Q was neither impaired in shedding activity nor in intracellular trafficking, as it was found on the cell membrane and active towards all tested substrates.

## 4. Discussion

Recent in vitro and mouse studies have shown the involvement of ADAM17 in inflammation and cancer pathways [12,43,51,52,53,64]. The role of ADAM17 in colon cancer is thought to be linked to ADAM17-mediated shedding of EGF-R activating ligands, as well as IL-6 trans-signaling, which is also promoted by ADAM17 via the shedding of IL-6R [43]. Upregulation of both pathways are hallmarks and high risk factors of colon cancer and their activation is mediated and regulated by ADAM17 [51], underlining its role in pathology.

In this study, we analyzed cancer-associated ADAM17 variants to gain further insight into the role of ADAM17 in disease pathways and the effect of these mutations on the protein. We screened multiple human cancer databases for variants found in tumor tissue of cancer patients [55,56,57,58,59,60]. Among this dataset, a large percentage (34.9%) of ADAM17 mutations was found in cancer of the gastrointestinal tract. Colon cancer-associated mutations were of especially high incidence, making up 13.1% of the total dataset and 37.7% of the GIT-associated subset. Another large subset of variants was found in lung cancer (10.3%), in which ADAM17 has also been implicated [65]. In this study, we chose four ADAM17 variants located within the catalytic domain for analysis: three were found in colon cancer samples (E319G, E406X, M435I) and one in pancreatic cancer (P417Q), all of them resulting in an amino acid change or the introduction of an early stop codon. We postulated that these mutations would affect ADAM17 activity due to being localized in the catalytic domain of the protein and their proximity to the active site.

Our results show that indeed, overall ADAM17 activity is significantly altered in the E319G, E406X and M435I variants, while the P417Q variant does not differ from the wild type. In case of the E406X and M435I mutation, we observed significantly reduced activity towards all tested substrates (TNFα, IL-6-R, AREG and a fluorogenic peptide). For the M435I variant, the data correspond with the findings of Perez et al. [61], supporting their hypothesis that this variant is impaired in shedding substrates due to the importance of the Met-turn structure for proteolytic activity. Interestingly, we could observe changes in the cellular trafficking of this variant. Although our biotinylation experiments show that the M435I variant still reaches the cell surface, we could observe an intracellular signal of ADAM17 divergent from the wild type. By utilizing immunofluorescence techniques (co-staining with ER marker PDI as well as cell surface GFP construct), the M435I seems to strongly localize around the perimeter of the cell. Since there was no significant difference of ADAM17 protein level on the cell surface after biotinylation in comparison to the wild type, the M435I seems to accumulate in other cell compartments, but outside of ER-structures.

The E406X mutant is a truncated ADAM17 variant, consisting of only the pro-domain and part of the catalytic domain. Due to the truncation, this variant is lacking the zinc binding motif, as well as all downstream domains including the transmembrane domain. To no surprise, this variant showed a complete lack of proteolytic activity towards all tested substrates. The lack of its transmembrane domain led to impaired intracellular transport and absence of cell surface expression. Immunofluorescence data suggest that this variant was mostly ER-localized. In co-expression analyses, where E406X was transfected together with a substrate, we could consistently observe lower expression levels of the co-transfected substrates (TNFα, IL-6R, AREG). It seems that in the context of overexpression, this variant influences overall protein expression, which could be explained by its accumulation inside the ER and a potential activation of protein degradation pathways, like for example the ERAD pathway [66].

Interestingly, the ADAM17 E319G variant exhibited intriguing results. Although showing significantly impaired activity towards TNFα, IL-6R and the TNFα-derived fluorogenic peptide, this variant was fully capable of shedding AREG. This indicates that the mutation does not directly affect catalytic activity, but rather substrate recognition and specificity, leading to selective inactivity towards certain substrates. Cellular localization of this variant is comparable to the wild type, although we observed a slight, non-significant decrease in surface localization and increase in ER co-localization compared to the wild type. In all conducted experiments, the pancreatic cancer-associated variant P417Q was indistinguishable from the wild type protein. Even though the amino acid change (position 417) is adjacent to the zinc binding motif (405–416), the change from proline to glutamine appears to have no significant effect on the enzymatic function in our experimental context.

Altogether, the colon cancer-associated ADAM17 mutations analyzed in this study either negatively affected shedding ability and/or intracellular trafficking. These findings are contradictory to the current dogma about the relationship between ADAM17 and cancer, in which an activation and upregulation of ADAM17 is thought to promote tumor development via EGF-R activation and IL-6 trans-signaling [41,43,51,52]. For the variants characterized in this study, their specific role in the development of cancer remains unclear. Most notably, no detailed information about the patient, its symptoms or the genetic background was available. Therefore, no statement can be made about whether these mutations are actually driver mutations and actively promote tumor development, or are rather passenger mutations that occurred by chance along other more detrimental mutations. It is, however, notable that almost all cancer-associated ADAM17 mutations analyzed in this study, as well as in our previous study [14], seem to have a negative effect of proteolytic activity in some way. It remains unknown whether this downregulation of ADAM17 activity can positively influence the development and progression of CRC. It is known that in the hypomorphic ADAM17^ex/ex^ mouse model, regeneration of the intestinal epithelium is severely compromised due to reduced EGF-R signaling, leading to significantly higher and more prolonged inflammation in a DSS-induced colitis model [12]. Since chronic inflammation is one of highest risk factors for developing CRC, further studies investigating the role of ADAM17 downregulation in intestinal regeneration and chronic inflammation, utilizing suitable animal models and more physiological experimental approaches, might shed more light on the complex role of ADAM17 in cancer pathology. Moreover, a better understanding of ADAM17 (dys)function within the development and progression of this devastating disease might reveal novel possible approaches for treatment and prevention.

## Supplementary Materials

Supplementary materials can be found at https://www.mdpi.com/2227-9059/8/11/463/s1.

## Abbreviations

ADAM17	A Disintegrin and Metalloproteinase 17
AREG	Amphiregulin
CD	catalytic domain
CRC	colorectal cancer
dKO	double knock-out
EGF-R	epidermal growth factor receptor
ELISA	Enzyme-Linked Immunosorbent Assay
ER	endoplasmatic reticulum
HEK	human embryonic kidney cells
IL-6R	interleukin-6 receptor
kDa	kilo Dalton
m	murine
PDI	protein disulfide isomerase
PMA	phorbol 12-myristate 13-acetate
sTNFα	soluble tumor necrosis factor alpha
wt	wild type
