# Supplementary material for: Functional Characterization of Colon-Cancer-Associated Variants in ADAM17 Affecting the Catalytic Domain
Source: Biomedicines. 2020 Oct 30;8(11):463. doi: 10.3390/biomedicines8110463 (PMC7692748; doi:10.3390/biomedicines8110463)
Supplement: Supplementary file 1 [file biomedicines-08-00463-s001.pdf]

Supplementary Figure 1

A

Domain	UniProt_AC	Gene_Name	Accession	Genome_Position	Position_N	Ref_N	Var_N	Position_A	Ref_A	Var_A	Polyphen_Pred	Cancer_Type	Source	
	P78536	ADAM17	NM_003183	chr2:9695722-9695722	13	C	T	5	L	F	benign	DOID:4362 / Cervical cancer [Cerca]	TCGA	
Signal peptide	P78536	ADAM17	NM_003183	chr2:9695720-9695720	15	C	T	5	L	L	-	DOID:11054 / Urinary bladder cancer [UBC]	ICGC	
	P78536	ADAM17	NM_003183	chr2:9695720-9695720	15	C	T	5	L	L	-	DOID:11054 / Urinary bladder cancer [UBC]	TCGA	
	P78536	ADAM17	NM_003183	chr2:9695720-9695720	15	C	T	5	L	L	-	DOID:11054 / Urinary bladder cancer [UBC]	COSMIC	
	P78536	ADAM17	NM_003183	chr2:9695705-9695705	30	C	A	10	S	R	possibly damaging	DOID:1993 / Rectum cancer [Recca]	COSMIC	
	P78536	ADAM17	NM_003183	chr2:9695662-9695662	73	G	A	25	G	S	benign	DOID:1793 / Pancreatic cancer [PACA]	TCGA	
	P78536	ADAM17	NM_003183	chr2:9683394-9683394	118	T	C	40	S	P	probably damaging	DOID:3571 / Liver cancer [Livca]	TCGA	
	P78536	ADAM17	NM_003183	chr2:9683393-9683393	119	C	G	40	S	X	-	DOID:263 / Kidney cancer [Kidca]	COSMIC	
	P78536	ADAM17	NM_003183	chr2:9683351-9683351	161	C	T	54	S	L	probably damaging	DOID:1324 / Lung cancer [Lunca]	IntOGen	
	P78536	ADAM17	NM_003183	chr2:9683313-9683313	199	G	A	67	E	K	probably damaging	DOID:1324 / Lung cancer [Lunca]	IntOGen	
	P78536	ADAM17	NM_003183	chr2:9683306-9683306	206	T	C	69	L	P	probably damaging	DOID:10534 / Stomach cancer [Stoca]	ICGC	
	P78536	ADAM17	NM_003183	chr2:9683306-9683306	206	T	C	69	L	P	probably damaging	DOID:10534 / Stomach cancer [Stoca]	TCGA	
	P78536	ADAM17	NM_003183	chr2:9676938-9676938	250	A	C	84	T	P	probably damaging	DOID:363 / Uterine cancer [Uteca]	COSMIC	
	P78536	ADAM17	NM_003183	chr2:9676938-9676938	250	A	C	84	T	P	probably damaging	DOID:363 / Uterine cancer [Uteca]	TCGA	
	P78536	ADAM17	NM_003183	chr2:9676906-9676906	282	C	G	94	F	L	benign	DOID:4362 / Cervical cancer [Cerca]	TCGA	
	P78536	ADAM17	NM_003183	chr2:9676905-9676905	283	A	C	95	K	Q	benign	DOID:1324 / Lung cancer [Lunca]	IntOGen	
	P78536	ADAM17	NM_003183	chr2:9676896-9676896	292	G	C	98	V	L	possibly damaging	DOID:10534 / Stomach cancer [Stoca]	ICGC	
	P78536	ADAM17	NM_003183	chr2:9676896-9676896	292	G	C	98	V	L	possibly damaging	DOID:10534 / Stomach cancer [Stoca]	TCGA	
	P78536	ADAM17	NM_003183	chr2:9676879-9676879	309	C	T	103	N	N	-	DOID:363 / Uterine cancer [Uteca]	IntOGen	
	P78536	ADAM17	NM_003183	chr2:9676879-9676879	309	C	T	103	N	N	-	DOID:363 / Uterine cancer [Uteca]	COSMIC	
	P78536	ADAM17	NM_003183	chr2:9676879-9676879	309	C	T	103	N	N	-	DOID:363 / Uterine cancer [Uteca]	TCGA	
	P78536	ADAM17	NM_003183	chr2:9676860-9676860	328	A	C	110	K	Q	benign	DOID:363 / Uterine cancer [Uteca]	TCGA	
	P78536	ADAM17	NM_003183	chr2:9676856-9676856	332	G	A	111	W	X	-	DOID:1521 / Cecum cancer [Cecca]	COSMIC	
	**P78536**	**ADAM17**	**NM_003183**	**chr2:9676856-9676856**	**332**	**G**	**A**	**111**	**W**	**X**	**-**	**DOID:219 / Colon cancer [Colca]**	**IntOGen**	**1) W111X**
	P78536	ADAM17	NM_003183	chr2:9676834-9676834	354	C	T	118	H	H	-	DOID:10534 / Stomach cancer [Stoca]	ICGC	
Prodomain	P78536	ADAM17	NM_003183	chr2:9676834-9676834	354	C	T	118	H	H	-	DOID:10534 / Stomach cancer [Stoca]	TCGA	
	P78536	ADAM17	NM_003183	chr2:9676833-9676833	355	G	A	119	V	M	probably damaging	DOID:0060119 / Pharynx cancer [Phaca]	IntOGen	
	P78536	ADAM17	NM_003183	chr2:9676833-9676833	355	G	A	119	V	M	probably damaging	DOID:11934 / Head and neck cancer [H&NC]	TCGA	
	P78536	ADAM17	NM_003183	chr2:9676045-9676045	368	C	A	123	P	H	benign	DOID:3571 / Liver cancer [Livca]	TCGA	
	P78536	ADAM17	NM_003183	chr2:9676042-9676042	371	A	T	124	D	V	probably damaging	DOID:1324 / Lung cancer [Lunca]	COSMIC	
	P78536	ADAM17	NM_003183	chr2:9676016-9676016	397	G	A	133	D	N	probably damaging	DOID:1793 / Pancreatic cancer [PACA]	TCGA	
	P78536	ADAM17	NM_003183	chr2:9668050-9668050	484	A	C	162	K	Q	probably damaging	DOID:3571 / Liver cancer [Livca]	TCGA	
	P78536	ADAM17	NM_003183	chr2:9668046-9668046	488	G	T	163	R	I	probably damaging	DOID:363 / Uterine cancer [Uteca]	COSMIC	
	P78536	ADAM17	NM_003183	chr2:9668046-9668046	488	G	T	163	R	I	probably damaging	DOID:363 / Uterine cancer [Uteca]	TCGA	
	P78536	ADAM17	NM_003183	chr2:9668023-9668023	511	G	T	171	D	Y	probably damaging	DOID:363 / Uterine cancer [Uteca]	COSMIC	
	P78536	ADAM17	NM_003183	chr2:9668023-9668023	511	G	T	171	D	Y	probably damaging	DOID:363 / Uterine cancer [Uteca]	TCGA	
	P78536	ADAM17	NM_003183	chr2:9668007-9668007	527	C	T	176	S	L	probably damaging	DOID:11934 / Head and neck cancer [H&NC]	TCGA	
	P78536	ADAM17	NM_003183	chr2:9668007-9668007	527	C	T	176	S	L	probably damaging	DOID:4362 / Cervical cancer [Cerca]	TCGA	
	P78536	ADAM17	NM_003183	chr2:9668005-9668005	529	C	T	177	R	C	probably damaging	DOID:1521 / Cecum cancer [Cecca]	COSMIC	
	**P78536**	**ADAM17**	**NM_003183**	**chr2:9668005-9668005**	**529**	**C**	**T**	**177**	**R**	**C**	**probably damaging**	**DOID:219 / Colon cancer [Colca]**	**ICGC**	**2) R117C**
	P78536	ADAM17	NM_003183	chr2:9668005-9668005	529	C	T	177	R	C	probably damaging	DOID:219 / Colon cancer [Colca]	TCGA	
	P78536	ADAM17	NM_003183	chr2:9667999-9667999	535	C	T	179	Q	X	-	DOID:4362 / Cervical cancer [Cerca]	TCGA	
	P78536	ADAM17	NM_003183	chr2:9667992-9667992	542	C	T	181	P	L	probably damaging	DOID:4362 / Cervical cancer [Cerca]	TCGA	
	P78536	ADAM17	NM_003183	chr2:9667941-9667941	593	G	A	198	G	E	probably damaging	DOID:3571 / Liver cancer [Livca]	TCGA	
	P78536	ADAM17	NM_003183	chr2:9667935-9667935	599	T	C	200	V	A	benign	DOID:363 / Uterine cancer [Uteca]	COSMIC	
	P78536	ADAM17	NM_003183	chr2:9667935-9667935	599	T	C	200	V	A	benign	DOID:363 / Uterine cancer [Uteca]	TCGA	
	P78536	ADAM17	NM_003183	chr2:9666362-9666362	631	C	T	211	R	X	-	DOID:1993 / Rectum cancer [Recca]	COSMIC	
	P78536	ADAM17	NM_003183	chr2:9666322-9666322	671	C	T	224	T	M	probably damaging	DOID:10534 / Stomach cancer [Stoca]	ICGC	
	P78536	ADAM17	NM_003183	chr2:9666322-9666322	671	C	T	224	T	M	probably damaging	DOID:10534 / Stomach cancer [Stoca]	TCGA	
	P78536	ADAM17	NM_003183	chr2:9666321-9666321	672	G	T	224	T	T	-	DOID:1324 / Lung cancer [Lunca]	COSMIC	
	P78536	ADAM17	NM_003183	chr2:9666321-9666321	672	G	T	224	T	T	-	DOID:1324 / Lung cancer [Lunca]	IntOGen	
	P78536	ADAM17	NM_003183	chr2:9666299-9666299	694	G	C	232	D	H	probably damaging	DOID:11934 / Head and neck cancer [H&NC]	TCGA	
	P78536	ADAM17	NM_003183	chr2:9666292-9666292	701	G	A	234	R	H	probably damaging	DOID:1521 / Cecum cancer [Cecca]	COSMIC	
	P78536	ADAM17	NM_003183	chr2:9666288-9666288	705	C	G	235	F	L	probably damaging	DOID:1324 / Lung cancer [Lunca]	COSMIC	
	P78536	ADAM17	NM_003183	chr2:9666273-9666273	720	C	T	240	G	G	-	DOID:0060119 / Pharynx cancer [Phaca]	IntOGen	
	P78536	ADAM17	NM_003183	chr2:9666273-9666273	720	C	T	240	G	G	-	DOID:11934 / Head and neck cancer [H&NC]	TCGA	
	P78536	ADAM17	NM_003183	chr2:9666271-9666271	722	G	A	241	R	K	benign	DOID:1324 / Lung cancer [Lunca]	COSMIC	
	P78536	ADAM17	NM_003183	chr2:9666271-9666271	722	G	A	241	R	K	benign	DOID:1324 / Lung cancer [Lunca]	IntOGen	
	P78536	ADAM17	NM_003183	chr2:9663433-9663433	788	G	A	263	R	Q	possibly damaging	DOID:363 / Uterine cancer [Uteca]	TCGA	
	P78536	ADAM17	NM_003183	chr2:9661441-9661441	848	G	A	283	R	H	probably damaging	DOID:5041 / Esophageal cancer [EC]	COSMIC	
	P78536	ADAM17	NM_003183	chr2:9661438-9661438	851	T	G	284	I	S	probably damaging	DOID:2394 / Ovarian cancer [OVCA]	COSMIC	
	P78536	ADAM17	NM_003183	chr2:9661434-9661434	855	C	G	285	L	L	-	DOID:10283 / Prostate cancer [PCa]	COSMIC	
	P78536	ADAM17	NM_003183	chr2:9661434-9661434	855	C	T	285	L	L	-	DOID:11934 / Head and neck cancer [H&NC]	TCGA	
	P78536	ADAM17	NM_003183	chr2:9661401-9661401	888	G	A	296	K	K	-	DOID:1324 / Lung cancer [Lunca]	COSMIC	
	**P78536**	**ADAM17**	**NM_003183**	**chr2:9661333-9661333**	**956**	**A**	**G**	**319**	**E**	**G**	**probably damaging**	**DOID:219 / Colon cancer [Colca]**	**COSMIC**	**3) E319G**
	P78536	ADAM17	NM_003183	chr2:9661333-9661333	956	A	G	319	E	G	probably damaging	DOID:219 / Colon cancer [Colca]	ICGC	
	P78536	ADAM17	NM_003183	chr2:9661333-9661333	956	A	G	319	E	G	probably damaging	DOID:219 / Colon cancer [Colca]	TCGA	
	P78536	ADAM17	NM_003183	chr2:9658364-9658364	970	G	A	324	D	N	possibly damaging	DOID:1319 / Brain cancer [Braca]	COSMIC	
	P78536	ADAM17	NM_003183	chr2:9658327-9658327	1007	A	T	336	H	L	probably damaging	DOID:1324 / Lung cancer [Lunca]	COSMIC	
	P78536	ADAM17	NM_003183	chr2:9658327-9658327	1007	A	T	336	H	L	probably damaging	DOID:1324 / Lung cancer [Lunca]	COSMIC	
	P78536	ADAM17	NM_003183	chr2:9658312-9658312	1022	A	C	341	Q	P	probably damaging	DOID:1612 / Breast cancer [BRCA]	IntOGen	
Catalytic domain	P78536	ADAM17	NM_003183	chr2:9658312-9658312	1022	A	C	341	Q	P	probably damaging	DOID:1612 / Breast cancer [BRCA]	ICGC	
	P78536	ADAM17	NM_003183	chr2:9658312-9658312	1022	A	C	341	Q	P	probably damaging	DOID:1612 / Breast cancer [BRCA]	COSMIC	
	P78536	ADAM17	NM_003183	chr2:9658310-9658310	1024	G	T	342	D	Y	probably damaging	DOID:363 / Uterine cancer [Uteca]	COSMIC	
	P78536	ADAM17	NM_003183	chr2:9658310-9658310	1024	G	T	342	D	Y	probably damaging	DOID:363 / Uterine cancer [Uteca]	TCGA	
	P78536	ADAM17	NM_003183	chr2:9658248-9658248	1086	A	G	362	G	G	-	DOID:219 / Colon cancer [Colca]	COSMIC	
	P78536	ADAM17	NM_003183	chr2:9658248-9658248	1086	A	G	362	G	G	-	DOID:219 / Colon cancer [Colca]	ICGC	
	P78536	ADAM17	NM_003183	chr2:9658248-9658248	1086	A	G	362	G	G	-	DOID:219 / Colon cancer [Colca]	TCGA	
	P78536	ADAM17	NM_003183	chr2:9658104-9658104	1117	G	A	373	V	I	benign	DOID:1793 / Pancreatic cancer [PACA]	TCGA	
	P78536	ADAM17	NM_003183	chr2:9658087-9658087	1134	C	A	378	I	I	-	DOID:363 / Uterine cancer [Uteca]	COSMIC	
	P78536	ADAM17	NM_003183	chr2:9658087-9658087	1134	C	A	378	I	I	-	DOID:363 / Uterine cancer [Uteca]	TCGA	
	P78536	ADAM17	NM_003183	chr2:9658057-9658057	1164	G	T	388	K	N	probably damaging	DOID:363 / Uterine cancer [Uteca]	TCGA	
	P78536	ADAM17	NM_003183	chr2:9658057-9658057	1164	G	T	388	K	N	probably damaging	DOID:363 / Uterine cancer [Uteca]	COSMIC	
	**P78536**	**ADAM17**	**NM_003183**	**chr2:9650236-9650236**	**1216**	**G**	**T**	**406**	**E**	**X**	**-**	**DOID:219 / Colon cancer [Colca]**	**COSMIC**	**4) E406X**
	**P78536**	**ADAM17**	**NM_003183**	**chr2:9650202-9650202**	**1250**	**C**	**T**	**417**	**P**	**L**	**probably damaging**	**DOID:5041 / Esophageal cancer [EC]**	**ICGC**	***12) P417Q (control)***
	P78536	ADAM17	NM_003183	chr2:9650202-9650202	1250	C	A	417	P	Q	probably damaging	DOID:1793 / Pancreatic cancer [PACA]	TCGA	
	P78536	ADAM17	NM_003183	chr2:9650168-9650168	1284	C	T	428	D	D	-	DOID:363 / Uterine cancer [Uteca]	TCGA	
	P78536	ADAM17	NM_003183	chr2:9650168-9650168	1284	C	T	428	D	D	-	DOID:363 / Uterine cancer [Uteca]	COSMIC	
	P78536	ADAM17	NM_003183	chr2:9650164-9650164	1288	G	A	430	G	R	probably damaging	DOID:5041 / Esophageal cancer [EC]	ICGC	
	**P78536**	**ADAM17**	**NM_003183**	**chr2:9650147-9650147**	**1305**	**G**	**A**	**435**	**M**	**I**	**benign**	**DOID:219 / Colon cancer [Colca]**	**COSMIC**	**5) M435I**
	P78536	ADAM17	NM_003183	chr2:9650147-9650147	1305	G	A	435	M	I	benign	DOID:219 / Colon cancer [Colca]	IntOGen	
	P78536	ADAM17	NM_003183	chr2:9645487-9645487	1352	C	T	451	S	L	probably damaging	DOID:4362 / Cervical cancer [Cerca]	COSMIC	
	P78536	ADAM17	NM_003183	chr2:9645487-9645487	1352	C	T	451	S	L	probably damaging	DOID:4362 / Cervical cancer [Cerca]	TCGA	
	P78536	ADAM17	NM_003183	chr2:9645471-9645471	1368	A	G	456	Q	Q	-	DOID:263 / Kidney cancer [Kidca]	COSMIC	
	P78536	ADAM17	NM_003183	chr2:9645471-9645471	1368	A	G	456	Q	Q	-	DOID:263 / Kidney cancer [Kidca]	TCGA	
	P78536	ADAM17	NM_003183	chr2:9645420-9645420	1419	C	T	473	R	R	-	DOID:4362 / Cervical cancer [Cerca]	COSMIC	
	P78536	ADAM17	NM_003183	chr2:9645420-9645420	1419	C	T	473	R	R	-	DOID:4362 / Cervical cancer [Cerca]	TCGA	
	P78536	ADAM17	NM_003183	chr2:9645412-9645412	1427	A	C	476	K	T	benign	DOID:263 / Kidney cancer [Kidca]	ICGC	
	P78536	ADAM17	NM_003183	chr2:9645412-9645412	1427	A	C	476	K	T	benign	DOID:263 / Kidney cancer [Kidca]	TCGA	
	P78536	ADAM17	NM_003183	chr2:9645344-9645344	1495	G	A	499	D	N	probably damaging	DOID:1324 / Lung cancer [Lunca]	ICGC	
	P78536	ADAM17	NM_003183	chr2:9645344-9645344	1495	G	A	499	D	N	probably damaging	DOID:1324 / Lung cancer [Lunca]	COSMIC	
	P78536	ADAM17	NM_003183	chr2:9642395-9642395	1555	A	T	519	S	C	probably damaging	DOID:0060119 / Pharynx cancer [Phaca]	IntOGen	
	P78536	ADAM17	NM_003183	chr2:9642395-9642395	1555	A	T	519	S	C	probably damaging	DOID:11934 / Head and neck cancer [H&NC]	TCGA	
	P78536	ADAM17	NM_003183	chr2:9642368-9642368	1582	G	A	528	E	K	benign	DOID:11054 / Urinary bladder cancer [UBC]	IntOGen	
Disintegrin domain	P78536	ADAM17	NM_003183	chr2:9642368-9642368	1582	G	A	528	E	K	benign	DOID:11054 / Urinary bladder cancer [UBC]	ICGC	
	P78536	ADAM17	NM_003183	chr2:9642368-9642368	1582	G	A	528	E	K	benign	DOID:11054 / Urinary bladder cancer [UBC]	COSMIC	
	P78536	ADAM17	NM_003183	chr2:9642368-9642368	1582	G	A	528	E	K	benign	DOID:11054 / Urinary bladder cancer [UBC]	TCGA	
	**P78536**	**ADAM17**	**NM_003183**	**chr2:9642335-9642335**	**1615**	**A**	**G**	**539**	**N**	**D**	**benign**	**DOID:219 / Colon cancer [Colca]**	**COSMIC**	**6) N539D**
	P78536	ADAM17	NM_003183	chr2:9642317-9642317	1633	G	A	545	V	M	possibly damaging	DOID:1324 / Lung cancer [Lunca]	COSMIC	
	P78536	ADAM17	NM_003183	chr2:9642317-9642317	1633	G	A	545	V	M	possibly damaging	DOID:363 / Uterine cancer [Uteca]	COSMIC	
	P78536	ADAM17	NM_003183	chr2:9642317-9642317	1633	G	A	545	V	M	possibly damaging	DOID:1324 / Lung cancer [Lunca]	ICGC	
	P78536	ADAM17	NM_003183	chr2:9642317-9642317	1633	G	A	545	V	M	possibly damaging	DOID:363 / Uterine cancer [Uteca]	TCGA	

	P78536	ADAM17	NM_003183	chr2:9637359-9637359	1667	C	T	556	P	L	probably damaging	DOID:2394 / Ovarian cancer [OVCA]	COSMIC	
	P78536	ADAM17	NM_003183	chr2:9637328-9637328	1698	T	A	566	V	V	-	DOID:11934 / Head and neck cancer [H&NC]	TCGA	
Membrane-proximal domain	P78536	ADAM17	NM_003183	chr2:9637283-9637283	1743	C	T	581	F	F	-	DOID:11054 / Urinary bladder cancer [UBC]	COSMIC	
	P78536	ADAM17	NM_003183	chr2:9637283-9637283	1743	C	T	581	F	F	-	DOID:11054 / Urinary bladder cancer [UBC]	ICGC	
	P78536	ADAM17	NM_003183	chr2:9637283-9637283	1743	C	T	581	F	F	-	DOID:4362 / Cervical cancer [Cerca]	TCGA	
	P78536	ADAM17	NM_003183	chr2:9637283-9637283	1743	C	T	581	F	F	-	DOID:11054 / Urinary bladder cancer [UBC]	TCGA	
	P78536	ADAM17	NM_003183	chr2:9637279-9637279	1747	G	A	583	E	K	benign	DOID:363 / Uterine cancer [Uteca]	TCGA	
	P78536	ADAM17	NM_003183	chr2:9637279-9637279	1747	G	A	583	E	K	benign	DOID:363 / Uterine cancer [Uteca]	COSMIC	
	P78536	ADAM17	NM_003183	chr2:9634874-9634874	1806	G	C	602	V	V	-	DOID:10534 / Stomach cancer [Stoca]	ICGC	
	P78536	ADAM17	NM_003183	chr2:9634874-9634874	1806	G	C	602	V	V	-	DOID:10534 / Stomach cancer [Stoca]	TCGA	
	P78536	ADAM17	NM_003183	chr2:9634856-9634856	1824	T	C	608	S	S	-	DOID:4362 / Cervical cancer [Cerca]	TCGA	
	**P78536**	**ADAM17**	**NM_003183**	**chr2:9634834-9634834**	**1846**	**G**	**A**	**616**	**D**	**N**	**benign**	**DOID:219 / Colon cancer [Colca]**	**COSMIC**	**7) D616N**
	P78536	ADAM17	NM_003183	chr2:9634802-9634802	1878	A	G	626	K	K	-	DOID:3571 / Liver cancer [Livca]	COSMIC	
	P78536	ADAM17	NM_003183	chr2:9634802-9634802	1878	A	G	626	K	K	-	DOID:3571 / Liver cancer [Livca]	ICGC	
	**P78536**	**ADAM17**	**NM_003183**	**chr2:9634789-9634789**	**1891**	**A**	**G**	**631**	**T**	**A**	**benign**	**DOID:219 / Colon cancer [Colca]**	**COSMIC**	**8) T631A**
	P78536	ADAM17	NM_003183	chr2:9634779-9634779	1901	T	G	634	F	C	probably damaging	DOID:3571 / Liver cancer [Livca]	COSMIC	
	P78536	ADAM17	NM_003183	chr2:9634779-9634779	1901	T	G	634	F	C	probably damaging	DOID:3571 / Liver cancer [Livca]	ICGC	
	P78536	ADAM17	NM_003183	chr2:9634772-9634772	1908	C	T	636	D	D	-	DOID:1612 / Breast cancer [BRCA]	COSMIC	
	P78536	ADAM17	NM_003183	chr2:9634772-9634772	1908	C	T	636	D	D	-	DOID:1612 / Breast cancer [BRCA]	ICGC	
	P78536	ADAM17	NM_003183	chr2:9634772-9634772	1908	C	T	636	D	D	-	DOID:1612 / Breast cancer [BRCA]	TCGA	
	P78536	ADAM17	NM_003183	chr2:9633946-9633946	1923	T	C	641	C	C	-	DOID:1612 / Breast cancer [BRCA]	IntOGen	
	P78536	ADAM17	NM_003183	chr2:9633946-9633946	1923	T	C	641	C	C	-	DOID:1612 / Breast cancer [BRCA]	ICGC	
	P78536	ADAM17	NM_003183	chr2:9633946-9633946	1923	T	C	641	C	C	-	DOID:1612 / Breast cancer [BRCA]	TCGA	
	P78536	ADAM17	NM_003183	chr2:9633946-9633946	1923	T	C	641	C	C	-	DOID:1612 / Breast cancer [BRCA]	COSMIC	
	P78536	ADAM17	NM_003183	chr2:9633917-9633917	1952	G	A	651	R	Q	probably damaging	DOID:363 / Uterine cancer [Uteca]	COSMIC	
	P78536	ADAM17	NM_003183	chr2:9633917-9633917	1952	G	A	651	R	Q	probably damaging	DOID:1324 / Lung cancer [Lunca]	COSMIC	
	P78536	ADAM17	NM_003183	chr2:9633917-9633917	1952	G	A	651	R	Q	probably damaging	DOID:1324 / Lung cancer [Lunca]	IntOGen	
	P78536	ADAM17	NM_003183	chr2:9633917-9633917	1952	G	A	651	R	Q	probably damaging	DOID:363 / Uterine cancer [Uteca]	TCGA	
	P78536	ADAM17	NM_003183	chr2:9633904-9633904	1965	C	A	655	F	L	benign	DOID:11054 / Urinary bladder cancer [UBC]	COSMIC	
	P78536	ADAM17	NM_003183	chr2:9633904-9633904	1965	C	A	655	F	L	benign	DOID:11054 / Urinary bladder cancer [UBC]	ICGC	
	P78536	ADAM17	NM_003183	chr2:9633904-9633904	1965	C	A	655	F	L	benign	DOID:11054 / Urinary bladder cancer [UBC]	COSMIC	
	**P78536**	**ADAM17**	**NM_003183**	**chr2:9633899-9633899**	**1970**	**A**	**C**	**657**	**D**	**A**	**possibly damaging**	**DOID:219 / Colon cancer [Colca]**	**COSMIC**	**9) D657A**
	P78536	ADAM17	NM_003183	chr2:9633899-9633899	1970	A	C	657	D	A	possibly damaging	DOID:219 / Colon cancer [Colca]	ICGC	
	P78536	ADAM17	NM_003183	chr2:9633899-9633899	1970	A	C	657	D	A	possibly damaging	DOID:219 / Colon cancer [Colca]	TCGA	
Trans-membrane domain	P78536	ADAM17	NM_003183	chr2:9633093-9633093	2016	C	T	672	I	I	-	DOID:363 / Uterine cancer [Uteca]	COSMIC	
	P78536	ADAM17	NM_003183	chr2:9633093-9633093	2016	C	T	672	I	I	-	DOID:363 / Uterine cancer [Uteca]	IntOGen	
	P78536	ADAM17	NM_003183	chr2:9633093-9633093	2016	C	T	672	I	I	-	DOID:363 / Uterine cancer [Uteca]	TCGA	
	P78536	ADAM17	NM_003183	chr2:9633048-9633048	2061	C	T	687	F	F	-	DOID:1993 / Rectum cancer [Recca]	COSMIC	
	P78536	ADAM17	NM_003183	chr2:9633048-9633048	2061	C	T	687	F	F	-	DOID:1993 / Rectum cancer [Recca]	ICGC	
	P78536	ADAM17	NM_003183	chr2:9633048-9633048	2061	C	T	687	F	F	-	DOID:1993 / Rectum cancer [Recca]	TCGA	
Cytoplasmic region	P78536	ADAM17	NM_003183	chr2:9630624-9630624	2157	G	A	719	M	I	benign	DOID:263 / Kidney cancer [Kidca]	ICGC	
	P78536	ADAM17	NM_003183	chr2:9630624-9630624	2157	G	A	719	M	I	benign	DOID:263 / Kidney cancer [Kidca]	TCGA	
	P78536	ADAM17	NM_003183	chr2:9630613-9630613	2168	C	T	723	S	L	probably damaging	DOID:363 / Uterine cancer [Uteca]	COSMIC	
	P78536	ADAM17	NM_003183	chr2:9630613-9630613	2168	C	T	723	S	L	probably damaging	DOID:363 / Uterine cancer [Uteca]	TCGA	
	P78536	ADAM17	NM_003183	chr2:9630612-9630612	2169	G	T	723	S	S	-	DOID:10283 / Prostate cancer [PCa]	COSMIC	
	**P78536**	**ADAM17**	**NM_003183**	**chr2:9630607-9630607**	**2174**	**G**	**A**	**725**	**R**	**H**	**probably damaging**	**DOID:219 / Colon cancer [Colca]**	**COSMIC**	**10) R725H**
	**P78536**	**ADAM17**	**NM_003183**	**chr2:9630541-9630541**	**2240**	**C**	**T**	**747**	**S**	**L**	**benign**	**DOID:219 / Colon cancer [Colca]**	**ICGC**	**11) P783Q**
	P78536	ADAM17	NM_003183	chr2:9630541-9630541	2240	C	T	747	S	L	benign	DOID:219 / Colon cancer [Colca]	COSMIC	
	P78536	ADAM17	NM_003183	chr2:9630541-9630541	2240	C	T	747	S	L	benign	DOID:219 / Colon cancer [Colca]	TCGA	
	P78536	ADAM17	NM_003183	chr2:9630538-9630538	2243	C	T	748	A	V	benign	DOID:2394 / Ovarian cancer [OVCA]	COSMIC	
	P78536	ADAM17	NM_003183	chr2:9630433-9630433	2348	C	A	783	P	Q	probably damaging	DOID:219 / Colon cancer [Colca]	COSMIC	
	P78536	ADAM17	NM_003183	chr2:9630394-9630394	2387	C	T	796	T	M	probably damaging	DOID:3571 / Liver cancer [Livca]	TCGA	
	P78536	ADAM17	NM_003183	chr2:9630385-9630385	2396	C	T	799	P	L	possibly damaging	DOID:4159 / Skin cancer [Skica]	COSMIC	
	P78536	ADAM17	NM_003183	chr2:9630376-9630376	2405	G	A	802	R	K	possibly damaging	DOID:4159 / Skin cancer [Skica]	ICGC	
	P78536	ADAM17	NM_003183	chr2:9630376-9630376	2405	G	A	802	R	K	possibly damaging	DOID:4159 / Skin cancer [Skica]	TCGA	
	P78536	ADAM17	NM_003183	chr2:9630364-9630364	2417	C	T	806	A	V	possibly damaging	DOID:10534 / Stomach cancer [Stoca]	ICGC	
	P78536	ADAM17	NM_003183	chr2:9630364-9630364	2417	C	T	806	A	V	possibly damaging	DOID:10534 / Stomach cancer [Stoca]	TCGA	
	P78536	ADAM17	NM_003183	chr2:9630357-9630357	2424	C	A	808	S	S	-	DOID:10534 / Stomach cancer [Stoca]	ICGC	
	P78536	ADAM17	NM_003183	chr2:9630357-9630357	2424	C	A	808	S	S	-	DOID:10534 / Stomach cancer [Stoca]	TCGA	
	P78536	ADAM17	NM_003183	chr2:9630344-9630344	2437	C	T	813	R	C	probably damaging	DOID:0060119 / Pharynx cancer [Phaca]	IntOGen	
	P78536	ADAM17	NM_003183	chr2:9630344-9630344	2437	C	T	813	R	C	probably damaging	DOID:11934 / Head and neck cancer [H&NC]	TCGA	
	P78536	ADAM17	NM_003183	chr2:9630330-9630330	2451	T	C	817	V	V	-	DOID:10534 / Stomach cancer [Stoca]	ICGC	
	P78536	ADAM17	NM_003183	chr2:9630330-9630330	2451	T	C	817	V	V	-	DOID:10534 / Stomach cancer [Stoca]	TCGA	

B

	UniProt_AC	Gene_Name	Accession	Genome_Position	Position_N	Ref_N	Var_N	Position_A	Ref_A	Var_A	Polyphen_Pred	Cancer_Type	Source	Mutation:
Prodomain	P78536	ADAM17	NM_003183	chr2:9676856-9676856	332	G	A	111	W	X	-	DOID:219 / Colon cancer [Colca]	IntOGen	**1) W111X**
	P78536	ADAM17	NM_003183	chr2:9668005-9668005	529	C	T	177	R	C	probably damaging	DOID:219 / Colon cancer [Colca]	ICGC	**2) R117C**
Catalytic domain	P78536	ADAM17	NM_003183	chr2:9661333-9661333	956	A	G	319	E	G	probably damaging	DOID:219 / Colon cancer [Colca]	COSMIC	**3) E319G**
	P78536	ADAM17	NM_003183	chr2:9650236-9650236	1216	G	T	406	E	X	-	DOID:219 / Colon cancer [Colca]	COSMIC	**4) E406X**
	P78536	ADAM17	NM_003183	chr2:9650147-9650147	1305	G	A	435	M	I	benign	DOID:219 / Colon cancer [Colca]	COSMIC	**5) M435I**
Disintegrin domain	P78536	ADAM17	NM_003183	chr2:9642335-9642335	1615	A	G	539	N	D	benign	DOID:219 / Colon cancer [Colca]	COSMIC	**6) N539D**
Membrane-proximal domain	P78536	ADAM17	NM_003183	chr2:9634834-9634834	1846	G	A	616	D	N	benign	DOID:219 / Colon cancer [Colca]	COSMIC	**7) D616N**
	P78536	ADAM17	NM_003183	chr2:9634789-9634789	1891	A	G	631	T	A	benign	DOID:219 / Colon cancer [Colca]	COSMIC	**8) T631A**
	P78536	ADAM17	NM_003183	chr2:9633899-9633899	1970	A	C	657	D	A	possibly damaging	DOID:219 / Colon cancer [Colca]	COSMIC	**9) D657A**
Cytoplasmic region	P78536	ADAM17	NM_003183	chr2:9630607-9630607	2174	G	A	725	R	H	probably damaging	DOID:219 / Colon cancer [Colca]	COSMIC	**10) R725H**
	P78536	ADAM17	NM_003183	chr2:9630541-9630541	2240	C	T	747	S	L	benign	DOID:219 / Colon cancer [Colca]	ICGC	**11) P783Q**

Included as control:

Catalytic domain	P78536	ADAM17	NM_003183	chr2:9650202-9650202	1250	C	T	417	P	L	probably damaging	DOID:5041 / Esophageal cancer [EC]	ICGC	12) P417Q
